# Effects of Low/Medium-Intensity Exercise on Fat Metabolism after a 6-h Fast

**DOI:** 10.3390/ijerph192315502

**Published:** 2022-11-23

**Authors:** Ming-Yi Liu, Shung-Quan Chen

**Affiliations:** 1 Department of Senior Welfare and Services, Southern Taiwan University of Science and Technology, No. 1, Nan-Tai Street, Yungkang District, Tainan 710301, Taiwan; 2 Office of Student Affairs, Tainan City Siaying Elementary School, No. 72, Sect. 2, Jhongshan Rd., Siaying District, Tainan 73541, Taiwan

**Keywords:** substrate metabolism, fasted state, low and medium exercise intensity

## Abstract

The effects of fasting and different exercise intensities on lipid metabolism were investigated in 12 male students aged 19.9 ± 1.4 years, with maximal oxygen consumption (VO_2max_) of 50.33 ± 4.0 mL/kg/min, using a counterbalanced design. Each participant ran on a treadmill at 45% and 65% VO_2max_ continuously for 20 min, followed by running at 85% VO_2max_ for 20 min (or until exhaustion) under a fed or fasted state (6 h). The respiratory exchange ratio (RER), blood glucose (BGLU), blood lactate (BLA), and blood triglyceride (TG) were analyzed during exercise. The results showed that the intensity of exercise did not significantly affect the BGLU and TG in the fed state. The levels of both RER and BLA increased as the intensity of exercise increased from low to high (45, 65, and 85% VO_2max_), and more energy was converted from fat into glucose at a high intensity of exercise. In the fasted state of 6 h, the BGLU level increased parallel to the intensity of exercise. The RER was close to 1.0 at a high intensity of exercise, indicating that more energy was converted from glycogen. At the intensities of 45 and 65% VO_2max_, the RER and concentration of TG were both lower in the fasted than in the fed state, showing that a higher percentage of energy comes from fat than in the fed state at 45 and 65% VO_2max_. When running at 85% VO_2max_, the BGLU concentration was higher in the fasted than in the fed state, indicating that the liver tissues release more BGLU for energy in the fasted state. Therefore, in the fasted state, running at 45% and 65% of VO_2max_ significantly affects lipid metabolism. On the contrary, the higher RER and BGLU concentrations when running at 85% VO_2max_ revealed no significant difference between the two probes. This study suggests that medium- and low-intensity exercise (45 and 65% VO_2max_) in the fasted state enhances lipid metabolism.

## 1. Introduction

Lipids are the most efficient substrates for energy storage devised by nature. They have the highest energy yield of all the energy sources stored in the body. Nevertheless, they are harmful because many fats accumulate in the body. Interventions concerning exercise and nutrition are commonly used strategies to control weight. Exercise is used to increase energy consumption and fat utilization; nutritional interventions are used to reduce the intake of extra fat and control the balance of energy metabolism. Although the methods are different, the goals are the same: to reduce the accumulation of body fat and to improve chronic diseases that involve insulin resistance, glucose, and lipid metabolism [1,2].

The Respiratory Exchange Ratio (RER) is the ratio of the volume of CO_2_ being produced by the body to the amount of O_2_ being consumed. The RER is generally used to evaluate energy conversion as an estimate of the proportion of energy sources during exercise. However, the prediction of this method can only be used when the energy source is supplied by carbohydrates and fats, and RER cannot be determined if the energy source is mainly protein [3,4]. It has been demonstrated that exercise after fasting can stimulate more fat to be used from adipose tissue, improving metabolic problems [5,6,7]. Fasting is characterized by no energy intake for sustained time periods, ranging from a few hours to days [8]. As a fasting period continues to exceed the first few hours, substrate utilization is mainly transformed from glycogenolysis into the use of lipids for energy [9].

Exercise under short-term fasting for 12 h (ranging from 12 h to 20 h) increases lipolysis in adipose tissue while also stimulating peripheral fat oxidation, resulting in increased fat utilization and weight loss [10]. However, exercise under long-term fasting will result in the loss of muscle mass, and in sports performance, it will affect cardiopulmonary function, heart rate, and fatigue. The results of previous studies suggest that prolonged fasting (23 h to 4 days) stimulates the breakdown of adipose tissue during exercise, releasing more fatty acids into the bloodstream [11,12,13,14,15,16,17,18,19,20,21] and increasing the percentage of fatty acids used by muscles [12,13,14,18,19]. Additionally, it was found that exercise does not cause exhaustion due to insufficient blood glucose during exercise after undergoing long-term fasting [11,13,14,16,17,18]. However, many exercise physiologists later found that prolonged fasting affects endurance exercise performance [17,19,20] and increases an individual’s heart rate, oxygen uptake, the degree of conscious fatigue [12,14,19], other physiological and psychological loads, and even early exercise to exhaustion [17,19,20].

Fatty acids are the main source of energy in skeletal muscles during rest and mild-intensity exercise. Many nutritional strategies, such as high-fat diets, fasting, lipid injections, and some food and drug supplements, use fatty acids mainly to increase muscle triglycerides or free fatty acids in the blood, to increase fatty acids as an energy source, and to reduce sugar use (the theoretical “glycogen-sparing effect”) to improve athletic performance. Fasting is a common practice for weight loss, but it is detrimental to athletic endurance performance. Therefore, fasting is no longer recommended to improve endurance sport performance, unless certain weight-graded competitions require rapid weight loss strategies.

In real life, a fasting situation for 12 h or more before exercising may be too long for the average person, and it is not easy to implement. Previous research lacked studies of exercise after fasting for less than 12 h. Therefore, the purpose of this study was to investigate the effects of different exercise intensities (45%, 65%, and 85% maximal oxygen consumption) on lipid metabolism after fasting for 6 h in young men.

## 2. Materials and Methods

### 2.1. Subjects

A total of 12 healthy male student participants, with ages ranging from 18 to 22 years, volunteered for this study. The exclusion criteria were chronic alcohol consumption, smoking, metabolic or endocrine diseases, and use of any anti-inflammatory drug. Those subjects who failed to achieve maximal oxygen consumption (VO_2max_) were also excluded from the study. The subjects were required to complete a health questionnaire to identify those without diabetes and cardiovascular or other major diseases. The participants were asked to avoid strenuous exercise for more than 30 min to avoid pre-exercise energy consumption that can affect the performance of exercise tests when completed 48 h before the test. During the experiment, each participant was asked not to overeat, not to deliberately go on a diet, and not to deliberately take nutritional supplements. They were also asked not to drink any beverages containing alcoholic ingredients. This study was approved by the Ethics Committee of the National University of Tainan and Tainan Sin-Lau Hospital (grant no. SLH919-08). All the participants provided written, informed consent before data were collected. The subject-level data were anonymized and deidentified prior to analysis.

### 2.2. Exercise Trials

**Figure 1:**
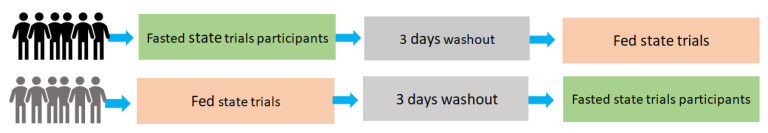
A counterbalanced design was applied, and, therefore, all participants completed both the fed and fasted training.

Before performing the trials, the participants completed 2 h per week of regular physical activity with a physical education teacher for 6 weeks. The participants were randomly assigned to two groups. Each subject underwent in fed and fasted states. The experiments were conducted with a counterbalanced measures design (Figure 1). The first trial was followed by a 3-day washout phase before the second trial started with a crossover test. Each participant ran on a treadmill at maximum oxygen uptakes of 45% and 65% VO_2max_ continuously for 20 min, followed by running at 85% VO_2max_ for 20 min (or until exhaustion) under the fed or fasted state (6 h). The respiratory exchange ratio (RER), blood glucose (BGLU), blood lactate (BLA), and blood triglyceride (TG) were analyzed during exercise. 

### 2.3. VO_2max_ Estimation before the Experiments 

Subjects had their VO_2max_ measured five days before the start of the experiments. The test was conducted on a treadmill (Quinton ST65, Seattle, WA, USA), and a portable metabolic analyzer (Cosmed K4b2, Rome, Italy) was used to evaluate the oxygen consumption (VO_2_), carbon dioxide production (VCO_2_), and RER. Prior to the incremental exercise test, a short walking warm-up (3 min) was carried out at 2.5 mph·h^−1^ with no inclination. The test started at 3 mph·h^−1^ at a 5% gradient. The velocity was then increased every 3 min by 1 mph·h^−1^ until exhaustion, following the American College of Sports Medicine general guidelines for stopping an exercise test [22]. The VO_2_, VCO_2_, heart rate, and perceived exertion on 0–10 Borg scales [23] were recorded.

### 2.4. Determination of VO_2max_

When a subject in this study reached any three of the following four criteria, it was determined that the subject reached their maximum oxygen uptake:

(1) When the intensity of the exercise load increases, the oxygen uptake no longer increases or the increase is less than 150 mL/min;
(2) The heart rate reaches ±10 beats/min of the maximum predicted value;
(3) The respiratory exchange rate is greater than 1.1;
(4) The ventilation volume per minute is more than 110 L.

The subject was re-measured for maximum oxygen uptake when fewer than three of the four criteria were reached.

### 2.5. The Treadmill Speed and the Grade

The non-maximal exercise load prediction formula of the subjects was established using the data obtained from the subjects after the maximal oxygen uptake test. The researchers obtained the oxygen uptake values (VO_2_) in the second and third minutes of each speed and constructed a regression equation with the speed of the treadmill. This equation was used as the non-maximal exercise load prediction formula for the subjects. The values obtained by multiplying the maximum oxygen uptake of the subjects by exercise intensities of 45%, 65%, and 85% VO_2max_ (%VO_2max_), were substituted into the exercise load prediction formula to calculate exercise speed converted from exercise intensity. 

### 2.6. Diet Controls

The fed group consumed a 500 kcal liquid diet (55% carbohydrates, 15% protein, and 35% fat) one hour before the experiment between 6 pm and 8 pm. After a short rest, the experimental treatment of exercise in the fed state was carried out. The fasted group, after having lunch between 11 am and 12 pm, maintained a fasted state for 6 h and performed the experimental treatment of the fasted-state exercise between 6 pm and 8 pm.

### 2.7. Sample Collection

**Figure 2:**
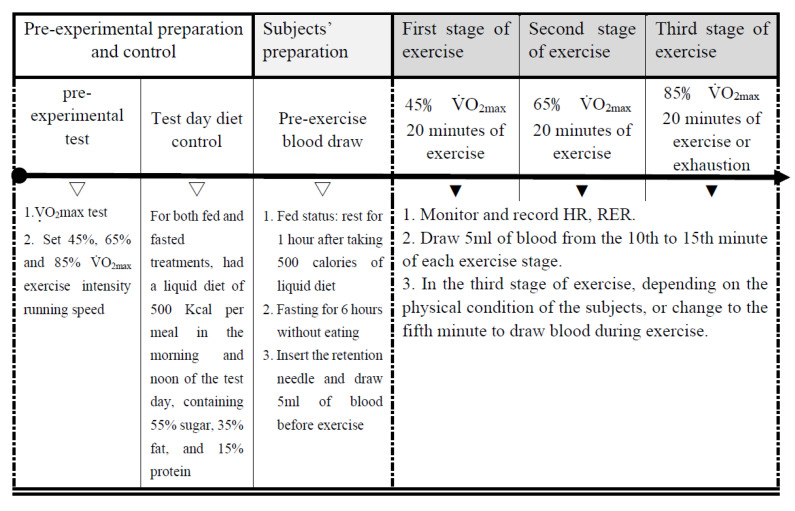
Procedure of the experiment.

Blood samples were drawn four times during each experimental treatment. Before the experiment, we inserted an indwelling needle into each subject’s elbow vein to collect 5 mL of blood. During the first and second stages of exercise at 45% and 65% VO_2max_, after 10 to 15 min of each stage (ensuring that the subject had entered a steady state), another 5 mL of blood was collected. When entering the third stage of exercise at 85% VO_2max_, if physiological indicators and the subject’s response (heartbeat rate, ventilation volume, and self-conscious effort) indicated that the exercise could not continue until the prescribed blood draw time, then the exercise was changed to the fifth-minute blood draw (Figure 2).

### 2.8. Statistical Analysis

Data were analyzed using SPSS version 17.0 (IBM Corp., Armonk, NY, USA). An a priori power analysis with an alpha of 0.05, a beta of 0.80, and an effect size of 0.8 estimated that 12 subjects were needed to achieve a power of 0.84. A two-way ANOVA for dependent samples was used, with the post hoc Bonferroni test used to analyze changes in the intragroup results. A significant probability level of 0.05 was used throughout the analyses. Data are presented as mean ± SD.

## 3. Results

Twelve male students participated in the experiment. No participants dropped out, and all participants completed the trial. The demographics and baseline characteristics are described in Table 1.

**Figure 3:**
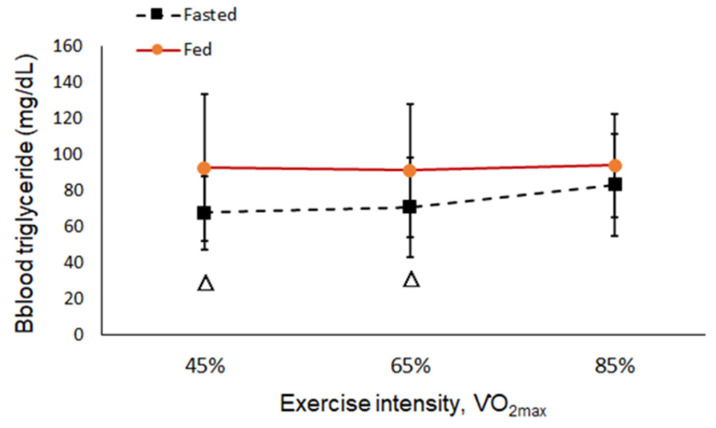
Blood triglyceride (TG) at each intensity in the fed and fasted state. “△”: Significant difference between the fed and fasted state at the same exercise intensity (*p* < 0.05).

**Figure 4:**
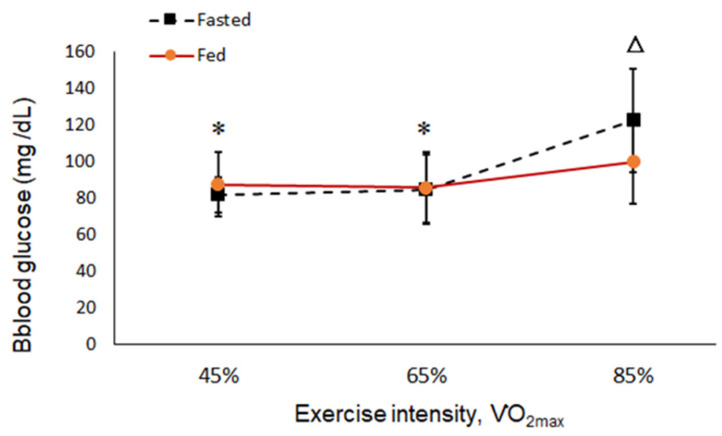
Blood glucose concentration (BGLU) at each exercise intensity in the fed and fasted state. “△”: Significant difference between the fed and fasted state at the same intensity (*p* < 0.05). “*”: Significant difference compared to 85% VO_2max_ in the same state (*p* < 0.05).

**Figure 5:**
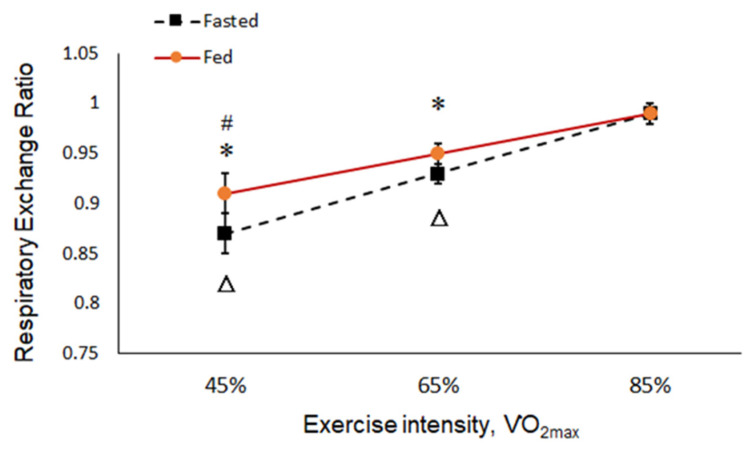
Respiratory exchange ratio (RER) at each intensity in the fed and fasted state. “*”: Significant difference compared to 85% VO_2max_ at 65% and 45% VO_2max_ in the same state (*p* < 0.05). “△”: Significant difference between the fed and fasted state at the same exercise intensity (*p* < 0.05). “#”: Significant difference compared to 65% VO_2max_ at 45% VO_2max_ in the same state (*p* < 0.05).

**Figure 6:**
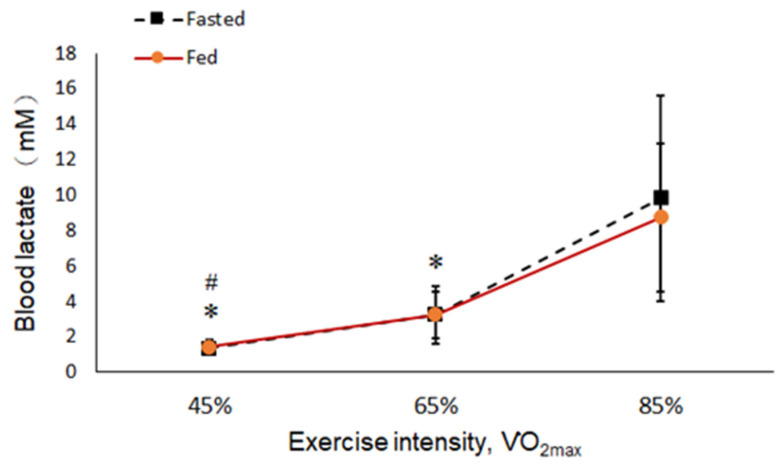
Blood lactate (BLA) at each intensity in the fed and fasted state. “*”: Significant difference compared to 85% VO_2max_ at 65% and 45% VO_2max_ in the same state (*p* < 0.05). “#”: Significant difference compared to 65% VO_2max_ at 45% VO_2max_ in the same state (*p* < 0.05).

The results showed metabolic changes with increasing exercise intensity. The concentration of TG increased with exercise intensity in the fasted and fed states, but there was no significant difference (Figure 3). In the 6-hour fasted state, the levels of BGLU increased significantly parallel to the intensity of the exercise (Figure 4). The levels of both RER and BLA increased as the intensity of exercise increased from low to high (45, 65, and 85% VO_2max_), and more energy was converted from fat into glucose at a high intensity of exercise regardless of a fed or fasted state (Figure 5 and Figure 6).

Table 2 shows a comparison of the differences between the fed and fasted probes. The RER was close to 1.0 at a high intensity of exercise, which indicated that more energy was converted from fat into glucose. At intensities of 45% and 65% VO_2max_, the RER and concentration of TG were both lower in the fasted than in the fed state (*p* < 0.05), where a lower RER indicated that a higher percentage of energy comes from fat in the fasted state at 45% and 65% VO_2max_. When running at 85% VO_2max_, the concentration of BGLU was higher in the fasted than in the fed state (*p* < 0.05), indicating that tissues release more BGLU for energy in the fasted state (Table 2). Therefore, in the fasted state, running at 45% and 65% VO_2max_ significantly affects lipid metabolism. On the contrary, the higher BGLU and BLA concentrations when running at 85% VO_2max_ revealed no obvious influence on lipid metabolism.

## 4. Discussion

We investigated the effects of exercise at different intensities after 6 h of fasting. The RER was close to 1.0 at a high intensity of exercise, which indicated that more energy was converted from fat to glucose. At the intensities of 45% and 65% VO_2max_, the RER and concentration of TG were both lower in the fasted than in the fed state, which indicated that a higher percentage of energy comes from fat at 45 and 65% VO_2max_. Therefore, in the fasted state, running at 45 and 65% VO_2max_ significantly affects lipid metabolism.

After fasting for 6 h, the BGLU of running at 85% VO_2max_ was significantly higher than that at 65% and 45% VO_2max_. There were no significant differences between 65% and 45% VO_2max_. These results may be explained by two reasons: (a) increased blood glucose from the liver in the fasted state during high-intensity exercise [24] or (b) increased blood glucose to the muscle in the fed state. The former hypothesis may indicate that the fasted state is more stressed during high-intensity exercise, resulting in a stronger hormonal response in the body. We think that the second reason is that the subjects in the fed state are affected by postprandial insulin and the amount of blood glucose entering the muscles increases during high-intensity exercise, which lowers the blood glucose level during exercise. Our rationale is based on the difference in blood glucose levels before exercise versus 45% VO_2max_ during exercise. The blood glucose in the fed state before exercise was higher than in the fasted state. If high blood glucose results in an insulin response (there were no measured insulin data in this study), then this possibility could explain the drop in blood glucose when subjects in the fed state start exercising. In addition, as other studies have demonstrated, the glucose transporter type 4 (GLUT-4) in muscle cells continues to function even when insulin concentrations return to baseline values in subjects who eat before exercise [25,26].

Blood glucose is the main source of carbohydrate energy at low exercise intensity. After gradually increasing exercise intensity, due to the action of hormones, the source of carbohydrates progressively depends on glycolysis in skeletal muscle and then glucose from the liver (hepatic glycogenolysis and gluconeogenesis). In addition to maintaining blood glucose levels, blood glucose gradually becomes an important source of carbohydrates for muscles. Although there are different conclusions about whether carbohydrate intake before exercise can cause an insulin response, it is inferred from the above that if the fed state before exercise causes the continuation of an insulin-related response, coupled with the addition of a hormonal stimulation response during high-intensity exercise, the blood sugar may show a low result. However, our study is limited to the analysis of collected data and can only make inferences from the monitored experimental variables. Perhaps more experimental variables, such as hormones, enzymes, etc., can be collected in future experiments for better exploration.

The phenomenon that the concentration of blood lactate rose sharply from 65% VO_2max_ to 85% VO_2max_ indicated that the energy system has largely relied on the production capacity of liver glucose metabolism. In this regard, the anaerobic metabolite of glucose is lactate, which will cause its concentration to increase significantly. This situation could cause body acidosis, which affects physiological metabolism.

Under the same exercise intensity, the RERs of 45% and 65% VO_2max_ in the fasted state were significantly lower than those in the fed state. There was no difference between the two probes under 85% VO_2max_. The respiratory exchange ratio is the proportion of carbon dioxide emitted by the individual to the oxygen used (VCO_2_/VO_2_). It is the proportion of energy source types in a steady state. The RER was between 0.7 and 1.0. A lower RER suggests a higher proportion of fat is used, whereas a higher value implies a higher proportion of carbohydrates is used. Our study has the same results as previous studies [12,13,27], which found that the fasted state uses more fat than the fed state.

Fasting before exercise may be used to increase the proportion of fat metabolism and to lose weight, but the effect is not obvious in the long term [28]. Additionally, past research has shown that fasting before exercise can improve insulin sensitivity [29,30]. However, previous studies lacked an investigation of exercise after fasting for less than 12 h. Our research has verified that fasting for 6 h can also increase fat metabolism, which serves as a reference for exercise program design.

## 5. Conclusions

Engaging in low- to medium-intensity exercise in a fasted state consumed more fat than doing so in a fed state, whereas high-intensity exercise did not show this phenomenon. During high-intensity exercise in a fed state, the blood glucose concentration will be significantly lower than that in a fasted state. This may be explained by the continuation of the insulin response caused by the diet and the additive effect of hormone stimulation during high-intensity exercise. There have been many previous exercise studies on fasting for 12 h (overnight) but exercising on an empty stomach before breakfast is not easy for many people. This study indicates that those who want to increase body fat consumption through exercise can arrange for moderate- and low-intensity exercise after fasting for 6 h. The results also suggest that exercise before dinner may be an effective approach. Future research can further explore whether the insulin response after eating will affect the phenomenon of blood sugar regulation during exercise.

## Figures and Tables

**Table 1:** Subject Characteristics (*n* = 12).

Age, Years	Hight, cm	Wight, kg	VO_2max_, mL·kg^−1^·min^−1^
19.9 ± 1.4	175 ± 3.1	70.1 ± 7.6	50.33 ± 4.0

**Table 2:** Respiratory exchange ratio (RER), blood glucose (BGLU), blood lactate (BLA), and blood triglyceride (TG) values for the fed and fasting state at each intensity.

Dieted State Exercise Intensity, VO_2max_		Fasted State (*n* = 12)	Fed State (*n* = 12)	*p* Value
RER	Before exercise	0.74 ± 0.01	0.81 ± 0.01	≤0.001
	45%	0.87 ± 0.02	0.91 ± 0.02	≤0.001
	65%	0.93 ± 0.01	0.95 ± 0.01	≤0.001
	85%	0.99 ± 0.01	0.99 ± 0.01	0.067
BLA (mM)	Before exercise	1.73 ± 0.62	2.18 ± 0.62	0.090
	45%	1.35 ± 0.35	1.44 ± 0.37	0.518
	65%	3.25 ± 1.62	3.25 ± 1.32	0.967
	85%	9.84 ± 5.81	8.75 ± 4.2	0.241
TG (mg/dL)	Before exercise	57.18 ± 13.4	67.63 ± 13.1	0.080
	45%	67.67 ± 20.1	92.67 ± 40.8	0.010
	65%	70.92 ± 27.4	91.17 ± 36.9	0.019
	85%x	83.33 ± 28.3	94.0 ± 28.5	0.250
BGLU (mg/dL)	Before exercise	79.3 ± 10.6	101.5 ±17.4	0.002
	45%	81.9 ± 9.5	87.42 ± 17.6	0.413
	65%	84.8 ± 19.2	85.8 ± 19.2	0.880
	85%	122.4 ± 28.0	99.7 ± 22.7	0.019

## Data Availability

The data used to support the findings of this study are available on request from the corresponding author.
